# Birth Type Lastingly Programs Secondary Hair Follicle Population and Cashmere Production in Cashmere Goats Despite Full Somatic Catch-Up Growth

**DOI:** 10.3390/ani16132018

**Published:** 2026-07-02

**Authors:** Kunjiang Li, Shaojie Wang, Zhenguo Wang, Xiujuan Jing, Zheqiao Zeng, Yanfang Shen, Guojing Jiang, Shuqi Liu, Chunxiang Zhang, Chunhe Yang, Youshe Ren

**Affiliations:** 1 College of Animal Science, Shanxi Agricultural University, Taigu 030801, China; 15309147519@163.com (K.L.); w3113693452@163.com (S.W.); wangzhenguo_0059@163.com (Z.W.); 15095486126@163.com (X.J.); 18696728136@163.com (Z.Z.); 15621938525@163.com (Y.S.); 18347570856@163.com (G.J.); 19970546113@163.com (S.L.); chunxiangzhang@sxau.edu.cn (C.Z.); 2 Key Laboratory of Farm Animal Genetic Resources Exploration and Breeding of Shanxi Province, Taigu 030801, China

**Keywords:** cashmere goat, litter size, secondary hair follicle, compensatory growth, cashmere yield, fiber diameter, follicle density

## Abstract

The objective of this study was to investigate the effects of birth type on growth performance, cashmere production traits, and hair follicle populations in cashmere goats. A total of 74 newborn kids (single, *n* = 38; twin, n = 36), all sired by a single Inner Mongolian Cashmere buck, were used in this study. Body weights were recorded at birth, weaning, and yearling age to calculate pre- and post-weaning average daily gain (ADG). At yearling age, cashmere samples and skin biopsies were collected for measurement of cashmere yield, fiber diameter, staple length, and primary and secondary hair follicle population parameters. Results showed that single-born kids exhibited significantly greater birth weight by 18.3% (2.85 ± 0.06 kg vs. 2.41 ± 0.06 kg), weaning weight by 10.3% (20.17 ± 0.32 kg vs. 18.28 ± 0.17 kg), pre-weaning weight gain by 9.2% (17.31 ± 0.31 kg vs. 15.85 ± 0.20 kg), and pre-weaning ADG by 14.5% (146.5 ± 3.4 g vs. 127.9 ± 1.6 g) than twin-born kids (*p* < 0.05). Conversely, twin-born kids surpassed single-born kids in post-weaning weight gain by 14.4% (14.60 ± 0.48 kg vs. 12.76 ± 0.48 kg) and post-weaning ADG by 16.8% (60.5 ± 1.9 g vs. 51.8 ± 1.9 g), indicative of clear compensatory growth, resulting in no significant difference in yearling weight (32.90 ± 0.51 kg). Birth type significantly affected yearling cashmere production: single-born kids produced 10.1% more greasy cashmere (810 ± 22 g vs. 736 ± 28 g; *p* = 0.046) and exhibited a 0.45 μm finer mean fiber diameter (14.34 ± 0.10 μm vs. 14.79 ± 0.11 μm; *p* = 0.003), whereas staple length did not differ between groups (9.70 ± 0.12 cm). Consistent with the fiber trait results, no differences were detected in any primary hair follicle population parameters. By contrast, all secondary hair follicle traits were significantly higher in single-born goats, with secondary follicle density (SFD) being 10.6% greater (36.6 ± 1.0 vs. 33.1 ± 0.8; *p* = 0.010), secondary follicle density index 11.7% greater (363.9 ± 9.4 vs. 325.7 ± 7.6; *p* = 0.003), secondary follicle number 12.4% greater (33.46 ± 0.87 vs. 29.78 ± 0.70; *p* = 0.002), and the S/P ratio 8.9% greater (12.11 ± 0.36 vs. 11.12 ± 0.29; *p* = 0.041). All secondary follicle population parameters were negatively correlated with fiber diameter, among which SFD showed the strongest association (r = −0.70, *p* < 0.001). These findings indicate that birth type is associated with lasting differences in secondary hair follicle endowment and cashmere production, which persist at least through yearling age despite full compensatory body growth in twin-born kids. The observed association is consistent with the hypothesis that the pre-weaning period constitutes a critical window for follicle maturation—a window during which secondary follicle development is highly sensitive to nutritional status during gestation and early postnatal life. This suggests that targeted nutritional management of twin-bearing does during late gestation and early lactation may represent a promising strategy to partially mitigate the disadvantage in cashmere production potential imposed by twinning. However, direct causal evidence for both the developmental programming hypothesis and the efficacy of such nutritional interventions requires further experimental validation, including longer-term studies tracking animals across multiple production cycles and controlled nutritional trials.

## Simple Summary

Cashmere goats often give birth to single or twin kids, and the number of kids born can influence their early growth and later productivity. This study investigated whether birth type—single or twin—affects growth, cashmere production, and the development of hair follicles in the skin. Single-born kids were heavier at birth and weaning and grew faster before weaning than twin-born kids. However, after weaning, twin-born kids grew more rapidly and caught up in body weight, so that by one year of age there was no difference in weight between the two groups. Despite this equal final body weight, single-born goats produced more cashmere (single-born group 810 ± 22 g; twin-born group 736 ± 28 g; 74 g higher yield, a 10.1% increase) and finer fibers (single-born group 14.34 ± 0.10 μm; twin-born group 14.79 ± 0.11 μm; 0.45 μm finer) than twin-born goats, while fiber staple length was similar. Examination of the skin showed that single-born goats had a greater density and number of secondary hair follicles—the structures that produce cashmere—whereas primary follicles were unaffected. All measures of secondary follicles were negatively correlated with fiber diameter, meaning that a higher follicle density is linked to finer cashmere. These findings indicate that, although twin-born kids achieved full compensatory body growth after weaning, the number of cashmere-producing secondary follicles—which, according to well-established developmental patterns, is permanently established by approximately three months of age—showed no evidence of a parallel compensatory increase. These findings, combined with the well-established sensitivity of secondary follicle maturation to nutritional status during gestation and early postnatal life, suggest that targeted gestational and early-lactation nutritional management of twin-bearing does—separating them from single-bearing does—may represent a promising strategy to partially mitigate the disadvantage in cashmere production potential imposed by twinning. This hypothesis awaits direct experimental verification.

## 1. Introduction

Cashmere, the fine undercoat produced by the secondary hair follicles (SHFs) of cashmere goats, is a luxury natural fiber prized for its exceptional fineness, softness, and thermal insulation. As a high-value commodity, it serves as an economic mainstay for pastoral communities across arid and semi-arid regions [1]. The commercial value of cashmere is governed primarily by two heritable quantitative traits—yield and fiber diameter—both of which are ultimately determined by the population and activity of SHFs. Histologically, total fiber output is a direct function of the absolute number of secondary follicles and the proportion that remain metabolically active [2]. Elucidating the factors that regulate SHF development and lifelong activity is therefore of both fundamental biological interest and practical importance for the genetic improvement and precision management of cashmere goats.

SHF morphogenesis and maintenance are orchestrated by a complex interplay of genetic programs and non-genetic factors. Among the latter, the intrauterine environment exerts a disproportionately formative influence through fetal programming, a process by which environmental cues during discrete critical windows induce persistent, often irreversible, modifications in tissue architecture and physiological function, largely via epigenetic mechanisms [3,4]. In cashmere goats, SHF initiation occurs predominantly during mid-to-late gestation, rendering this period acutely sensitive to intrauterine perturbations, particularly fluctuations in nutrient availability [5,6]. Birth type (singleton versus multiple) is a primary determinant of the prenatal environment, directly modulating fetal spatial distribution, nutrient partitioning, and endocrine milieu [7]. Compared with singletons, multiple fetuses experience heightened intrauterine competition and growth restriction, conditions that can impair follicle morphogenesis and permanently curtail follicle population and postnatal fiber-producing capacity [8]. Critically, unlike somatic tissues such as skeletal muscle and bone, which retain substantial capacity for compensatory growth following nutritional rehabilitation, SHF maturation is largely completed shortly after birth and remains fixed throughout life [9]. This fundamental biological dichotomy raises a compelling yet unresolved question: if multiple births cause early-life somatic growth retardation, can subsequent catch-up growth fully restore body mass while the architecture of cashmere-producing follicles remains lastingly compromised?

Contemporary breeding programs increasingly prioritize reproductive efficiency, driving an industry-wide shift toward higher kidding frequencies and the selection of prolific genotypes. Although economically attractive, this transition inevitably creates a critical tension between offspring number and individual fiber-producing capacity. Existing evidence on the effect of birth type on follicle development and downstream fiber traits remains fragmented and equivocal: while some studies have documented significant reductions in follicle population and cashmere yield in twin-born goats [10], others have found no clear associations [11], and none have systematically examined how whole-animal compensatory growth trajectories interact with follicle architecture to determine ultimate productivity. Consequently, whether the pursuit of prolificacy inadvertently penalizes lifetime cashmere output via developmental programming of SHFs remains a critical unresolved issue. Should such a trade-off exist, it would necessitate targeted early-life nutritional interventions to safeguard yield and quality while retaining the benefits of higher reproductive rates.

To address this knowledge gap, the present study was designed to evaluate the long-term effects of birth type (single vs. twin) on growth performance, cashmere production traits (yield, fiber diameter, and staple length), and follicle histological characteristics, with particular emphasis on the secondary follicle population. We hypothesized that birth type would be associated with differences in secondary follicle development and cashmere production at yearling age, with twin-born kids potentially showing lasting deficits despite post-weaning compensatory growth. The findings will provide a mechanistic framework for managing the trade-off between reproductive efficiency and cashmere quality, and will inform the design of breeding and nutritional strategies that maximize lifetime productivity in contemporary cashmere goat production systems.

## 2. Materials and Methods

### 2.1. Animals, Experimental Design, and Management

This study was approved by the Institutional Animal Care and Use Committee of Shanxi Agricultural University (Taigu, China; approval no. SXAU-EAW-2024S. YD. 001016478) and was conducted at the Yiwei White Cashmere Goat Co., Ltd. farm (39°11′ N, 107°16′ E) in the Inner Mongolia Autonomous Region, China. A total of 80 healthy female Inner Mongolian White Cashmere goat kids were used in this study. All kids were born to 3- to 4-year-old multiparous ewes between 10 and 17 March 2024, sired by a single Inner Mongolian Cashmere buck, and assigned to two groups according to birth type: a single-born group (*n* = 40, kids born as singletons) and a twin-born group (n = 40, kids born as twins). To balance maternal parity across groups, the dams of the single-born group comprised 15 third-parity and 25 fourth-parity ewes, whereas the twin-born group was derived from 10 third-parity and 10 fourth-parity ewes. The experimental animals were managed separately from the commercial flock. By weaning, two kids in the single-born group and four in the twin-born group had died due to diarrhea, yielding final group sizes of 38 and 36, respectively.

From birth to weaning, all kids and their dams were housed together as a single mixed group in an open-air pen (35 m × 20 m), with single- and twin-born kids managed under identical protocols throughout. During the first three days postpartum, kids remained continuously with their dams to ensure adequate colostrum intake. From day four onward, a twice-daily suckling regimen was implemented (07:00–08:00 and 17:00–18:00), after which kids were immediately separated from the dams. Ewes grazed on natural pasture following the morning suckling and were returned to the pen for rest after the afternoon suckling; kids remained in the open-air pen throughout the day. Creep feeding was initiated at 10 days of age: kids had ad libitum access to alfalfa hay and fresh water, and received a starter concentrate at an initial allowance of 25 g/kid/day, which was increased by 25 g/kid/day every two weeks until weaning. All kids were weaned at a uniform age of three months (90 ± 3 days), on the same calendar date. Throughout the pre-weaning period, pens were cleaned daily and thoroughly disinfected once a month. After weaning, kids were moved to a natural arid steppe pasture (desert steppe) for grazing and supplemented with 200 g/kid/day of maize until combing at 12 months of age.

### 2.2. Sample Collection

Body weight was recorded before the morning feeding at birth (0 d), weaning (3 mo), and 12 mo of age. At each time point, measurements were taken on two consecutive days, and were used for statistical analysis. Cashmere samples were collected at 13 mo of age (April of the following year), coinciding with the natural shedding period. A 5 cm × 5 cm area was delineated on the posterior edge of the left scapula, and all cashmere within this area was clipped flush with the skin surface using fine cashmere scissors. Samples were stored in sealed bags for subsequent determination of staple length and fiber diameter. Total cashmere yield was recorded at combing. Skin samples were obtained from the same anatomical site using a 1 cm diameter biopsy punch. Each specimen was immediately placed in a labeled cassette and fixed in 4% paraformaldehyde solution for 24 h prior to paraffin embedding. When bleeding occurred, hemostasis was achieved by applying pressure with sterile gauze; topical Yunnan Baiyao powder was applied when necessary.

### 2.3. Determination of Cashmere Staple Length and Fiber Diameter

Cashmere samples were first washed with distilled water, soaked overnight in carbon tetrachloride solution, and then air-dried at room temperature. Staple length was measured by placing individual fibers on a black velvet board, gently clamping both ends with fine tweezers, and slowly stretching each fiber until the crimp was fully eliminated; the extended length was recorded as the staple length. Two hundred fibers were measured per sample, and the arithmetic mean was calculated. Fiber diameter was determined using a CU-6 fiber fineness meter equipped with a micro-projection system (Beijing Unitedvision Technology Co., Ltd., Beijing, China). Fibers were cut at their midpoint with a Halberg microtome (Shanghai Biaozhuo Scientific Instruments Co., Ltd., Shanghai, China) to obtain 0.5 mm fragments, which were then mounted on a glass slide with a small amount of glycerol and covered with a coverslip. Diameter measurements were performed under a microscope. Two hundred fibers were measured per sample, and the arithmetic mean was taken as the mean fiber diameter.

### 2.4. Determination of Hair Follicle Number

Paraffin block preparation, hair follicle sectioning, and follicle counting were performed following the procedures of Yang et al. [12]. Briefly, skin samples were fixed in 4% paraformaldehyde for 24 h, dehydrated through a graded ethanol series, cleared in xylene, and embedded in paraffin. Transverse serial sections (5 μm thick) were cut at the level of the mid-sebaceous gland using a Leica rotary microtome and stained using the Sacpic method. Images were captured with a Leica ICC 50 W microscope (Leica, Wetzlar, Germany). For each sample, ten randomly selected, non-overlapping microscopic fields (1.11 mm^2^ per field) were photographed. The numbers of primary and secondary hair follicles were counted in each field, and the mean values were calculated as the primary follicle count and secondary follicle count for the individual sample. From these data, follicle density (follicles/mm^2^), the follicle density index, and the total follicle number per skin area were derived [12].

### 2.5. Statistical Analyses

All statistical analyses were performed using SAS version 9.4 (SAS Institute Inc., Cary, NC, USA). Prior to modelling, data were examined for normality and homoscedasticity of residuals via quantile–quantile plots, studentized residual plots, and the Shapiro–Wilk and Levene’s tests. No severe departures from model assumptions were detected for any trait; therefore, all variables were analyzed on the original measurement scale.

To account for the inherent non-independence of twin kids born to the same dam, a linear mixed-model framework was adopted. Models were fitted using the MIXED procedure with restricted maximum likelihood (REML). The fixed structure comprised birth type (single vs. twin), parity (third vs. fourth parity, included to adjust for the unequal parity distribution between single- and twin-bearing dams), and their interaction. Dam identity was included as a random intercept to model the covariance between siblings and to obtain correct standard errors for the fixed effects. The full model for each trait was:

Y*_ijk_* = μ + BirthType_*i*_ + Parity_j_ + (BirthType × Parity)_*ij*_ + Dam_*k*_ + ε_*ijk*_,

where Y*_ijk_* is the observation on the *k*th dam in the *ij* th birth-type × parity combination, μ is the overall mean, BirthType*_i_* and Parity*_j_* are fixed effects, Dam*_k_*~N (0, σ^2^_dam_) is the random dam effect, and ε*_ijk_*~N (0, σ^2^*_e_*) is the residual error.

The denominator degrees of freedom were approximated using the Kenward-Roger method to correct for the small-sample bias and the unbalanced data structure. For each trait, the birth-type × parity interaction was tested first. When the interaction was non-significant (*p* > 0.05), it was removed and the reduced main-effects model was refitted. Least squares mean (LSM) ± standard errors (SEM) for birth type were estimated from the final model, and pairwise comparisons were adjusted using the Tukey–Kramer method. All tests were two-sided, with statistical significance declared at *p* < 0.05. Exact *p*-values are reported throughout.

Relationships between secondary hair follicle traits and cashmere fiber diameter were examined by Pearson correlation coefficients computed across all animals, independent of treatment group. The strength of correlations was interpreted according to the following thresholds: |r| ≥ 0.50, strong; 0.30 ≤ |r| < 0.50, moderate; 0.10 ≤ |r| < 0.30, weak; and |r| < 0.10, negligible [13].

All results in the text, tables, and figures are presented as LSM ± SEM derived from the mixed models, unless otherwise noted.

## 3. Results

### 3.1. Effects of Birth Type on Growth Performance at Different Developmental Stages

Birth type significantly affected all growth traits examined (*p* < 0.05 or *p* < 0.01), with the sole exception of yearling weight (Table 1). Compared with twin-born kids, single-born kids were 18.3% heavier at birth and 10.3% heavier at weaning, and they had 9.2% greater pre-weaning weight gain and 14.5% greater pre-weaning average daily gain (ADG). In contrast, twin-born kids surpassed single-born kids in post-weaning weight gain by 14.4% and post-weaning ADG by 16.8%, indicating compensatory growth. Yearling weight averaged 32.90 ± 0.51 kg and did not differ significantly between birth types.

### 3.2. Cashmere Production and Quality of Yearling Goats

**Figure 1:**
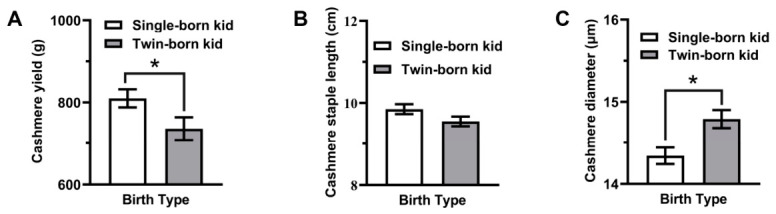
Effects of birth type on cashmere production and quality. (**A**) Cashmere yield; (**B**) Cashmere staple length; (**C**) Cashmer diameter. Data are presented as Least Squares Mean ± SEM. Single-born group: white bars; twin-born group: grey bars. * *p* < 0.05.

Birth type had a significant effect on yearling greasy cashmere yield and fiber diameter (*p* < 0.05), whereas fiber staple length was not affected (Figure 1). Single-born kids produced 10.1% more greasy cashmere (*p* = 0.046) and exhibited a 0.45 μm finer mean fiber diameter (*p* = 0.003) than twin-born kids. Fiber staple length averaged 9.70 ± 0.12 cm overall and did not differ between groups.

### 3.3. Hair Follicle Population

**Figure 2:**
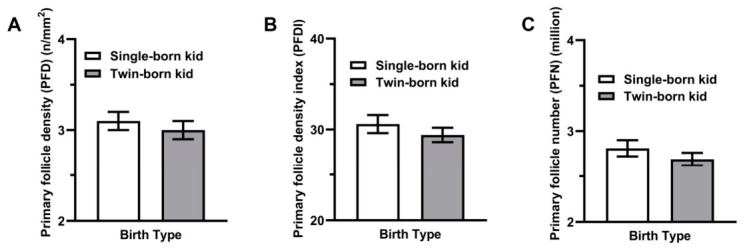
Effects of birth type on primary hair follicle traits. (**A**) Primary follicle density (PFD); (**B**) Primary follicle density index (PFDI); (**C**) Primary follicle number (PFN); Data are presented as Least Squares Mean ± SEM. Single-born group: white bars; twin-born group: grey bars.

**Figure 3:**
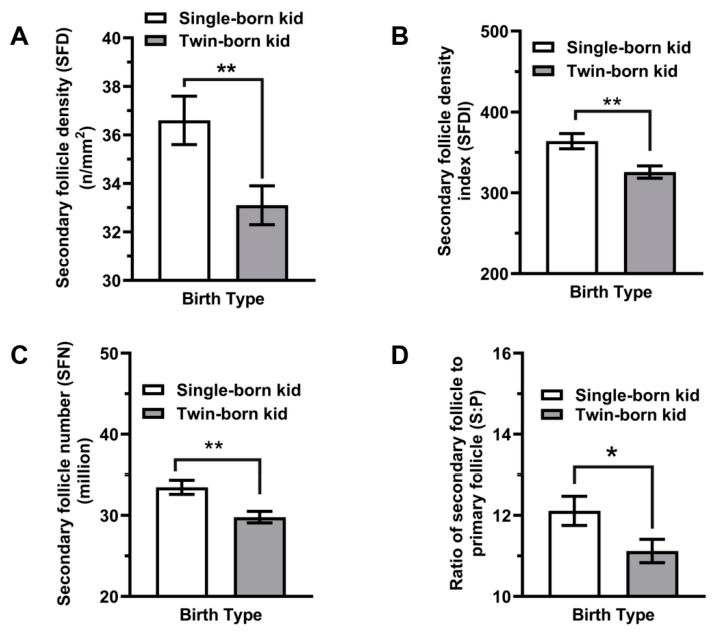
Effects of birth type on secondary hair follicle traits. (**A**) Secondary follicle density (SFD); (**B**) Secondary follicle density index (SFDI); (**C**) Secondary follicle number (SFN); (**D**) Ratio of Secondary follicle to primary follicle (S:P). Data are presented as Least Squares Mean ± SEM. Single-born group: white bars; twin-born group: grey bars. * *p* < 0.05, ** *p* < 0.01.

No significant differences were detected in primary follicle density (PFD), primary follicle density index (PFDI), or primary follicle number (PFN) between single- and twin-born goats; the overall means were 3.1 ± 0.1 follicles/mm^2^, 30.0 ± 0.9, and 2.75 ± 0.08, respectively (Figure 2). In marked contrast, single-born goats displayed significantly higher values for all secondary follicle parameters (Figure 3). At yearling age, secondary follicle density (SFD) was 3.5 follicles/mm^2^ higher in single-born goats (10.6%, *p* = 0.010). The secondary follicle density index (SFDI) and secondary follicle number (SFN) were 11.7% and 12.4% higher, respectively, in the single-born group. Additionally, the secondary-to-primary follicle ratio (S/P) was 0.99 units greater (8.9%) in single-born goats (*p* = 0.041).

### 3.4. Association Between Cashmere Fiber Diameter and Secondary Hair Follicle Populations

To determine whether secondary follicle endowment is quantitatively associated with fiber fineness at the individual level—beyond the group-level differences established above—we examined correlations between all secondary follicle parameters and mean fiber diameter. All secondary follicle traits were significantly and negatively correlated with cashmere fiber diameter. Secondary hair follicle density (SFD) exhibited the strongest association (r = −0.70, *p* < 0.001), followed by the secondary follicle density index (SFDI) and secondary follicle number (SFN) (both r = −0.57, *p* < 0.001), and the secondary-to-primary follicle ratio (S/P; r = −0.44, *p* = 0.007).

## 4. Discussion

Birth type is a primary determinant of early growth and nutrient allocation in goats, with single-born kids typically exhibiting superior pre-weaning growth performance [14]. However, whether these early-life differences translate into long-term alterations in cashmere production and the underlying follicle population remains poorly understood. Given that the morphogenesis and maturation of hair follicles—particularly secondary hair follicles (SHFs)—are established during the later stages of pregnancy and the early period after birth [9,15] and are highly sensitive to nutritional and physiological state [6,16], we systematically examined the effects of birth type on growth traits, cashmere yield and quality, and hair follicle populations in yearling goats. Our results showed that single-born kids were heavier at birth and weaning, yet twin-born kids exhibited clear compensatory post-weaning growth, resulting in equivalent yearling body weights. Critically, single-born kids produced significantly more and finer cashmere, a phenotypic advantage underpinned by consistently superior secondary follicle traits, including higher density, greater number, and an elevated secondary-to-primary follicle (S/P) ratio. Furthermore, all measured SHF traits were significantly negatively correlated with fiber diameter, with secondary follicle density (SFD) displaying the strongest association.

A large body of evidence from diverse sheep and goat breeds consistently demonstrates that single-born offspring are heavier than multiples at birth and weaning. Consistent with this pattern, single-born kids in our study had significantly 0.44 kg greater birth weight than twins, with reported differences across breeds and production systems ranging from 0.34 to 1.50 kg [17,18,19,20]. This birth weight advantage persisted into the pre-weaning phase, during which singletons consistently outperformed twins in weaning weight and pre-weaning average daily gain (ADG) [21,22,23]. Our data confirm this general trajectory. In contrast to reports documenting lasting body weight differences through 12 months of age [24,25], yearling weight in our trial did not differ between birth types, a finding consistent with [20] and with the suggestion by Mavrogenis [26] that under uniform post-weaning management, growth rates between birth types tend to converge.

The lower birth weight of twins can be explained in large part by prenatal constraints on nutrient partitioning. Ewes bearing multiple fetuses simultaneously face limitations in uterine space and placental nutrient transfer capacity [27,28]. Elevated maternal cortisol concentrations associated with multiple pregnancies have been proposed as an endocrine mechanism that restricts individual fetal growth to alleviate maternal metabolic burden [7,23,29]. Elevated plasma cortisol in twin offspring shortly after birth further suggests the fetal growth restriction experienced in utero [30]. This prenatal constraint is compounded during lactation, when twin kids face intensified competition for a finite milk supply, further depressing pre-weaning growth relative to singletons [31,32,33]. Thus, the weaning weight deficit observed in our study and widely reported in the literature reflects the combined influence of both intrauterine constraint and postnatal milk competition, although the relative contribution of each cannot be quantified in the present natural-suckling design.

The convergence of yearling body weights between single- and twin-born kids provides compelling evidence for compensatory growth once nutritional constraints are lifted. After weaning onto a common high-plane nutritional regimen, previously restricted twin kids exhibited accelerated growth rates, a phenomenon well documented in ruminant production. Indeed, several studies have shown that birth type exerts either a non-significant or reversed effect on post-weaning ADG, supporting the capacity for substantial catch-up growth [20,24,26]. The early initiation of solid feed intake by twin lambs, observed in certain behavioral studies [34], may represent an adaptive strategy that facilitates this transition. Collectively, these findings demonstrate that while fetal programming and milk competition establish divergent early growth trajectories, adequate post-weaning nutrition enables twin-born kids to fully express their somatic growth potential, thereby closing the body weight gap by yearling age. This outcome underscores the plasticity of developmental constraints on body mass and highlights compensatory growth as a key postnatal mechanism in small ruminants. Future studies integrating measurements of maternal endocrine profiles, placental efficiency, and post-weaning nutritional management will be essential for designing strategies to mitigate early growth penalties in multiple-born kids.

Extending beyond somatic growth, our study reveals that birth type exerts a lasting and economically significant impact on cashmere production. Single-born kids produced 74 g (10.1%) more greasy cashmere with a 0.45 μm (3.0%) finer mean fiber diameter, while staple length remained unaffected. These findings align with a recurring pattern in the literature: birth type predominantly influences fleece weight and fiber diameter, whereas staple length is largely conserved. For instance, single-born lambs and kids have been reported to exhibit higher wool or cashmere production [8,20] and finer fiber diameter [10,35], with staple length comparable to that of multiples. Our results, combined with the absence of differences in primary follicle traits, demonstrate unequivocally that the superior cashmere performance of singletons originates from a selective advantage in SHF endowment. Indeed, single-born goats exhibited significantly greater SFD, SFN, and S/P ratio, strongly corroborating earlier histological observations in Liaoning and Inner Mongolian goats [11,36]. The robust negative correlations between all SHF parameters and fiber diameter, with SFD displaying the strongest association (r = −0.70, *p* < 0.001), provide a direct structural mechanism linking greater follicle density to finer fibers.

This selective and lasting impact on SHFs finds a mechanistic explanation in the narrowly defined ontogenetic window of follicle maturation. Whereas primary follicles are fully established at birth, the majority of SHFs complete their maturation during a critical postnatal period spanning approximately 3 to 6 months of age, after which the total SHF number is fixed for life. This window coincides precisely with the suckling phase, during which twin kids experience pronounced nutritional deprivation—a consequence of both intrauterine growth restriction and intensified postnatal milk competition, as reflected in their lower birth and weaning weights. Because these two sources of nutritional stress operate concurrently in our natural suckling design, the relative contribution of prenatal programming versus postnatal undernutrition to the observed SHF deficit cannot be determined. Consequently, the developmentally plastic SHF population in twin kids may fail to attain its full potential density, leading to a lasting deficit in the physical machinery for fiber production. This early-life constraint stands in stark contrast to the trajectory of body weight, which twin kids fully compensate for by yearling age. We therefore identify a striking developmental dichotomy: whereas somatic tissues such as muscle and bone retain a robust capacity for catch-up growth, the morphogenesis of SHFs is constrained by a strict temporal deadline that, once passed, precludes any subsequent recovery regardless of whether the initial deficit originated prenatally or postnatally. A limitation of the present study is that the natural nursing design precludes a definitive separation of prenatal and postnatal effects. Twin kids experienced both intrauterine competition and elevated postnatal competition for milk. While the birth weight difference clearly establishes a prenatal component, the observed differences in pre-weaning growth reflect a combination of both. Notably, the pronounced compensatory growth of twin kids after weaning, when the postnatal nutritional constraint was removed, provides suggestive evidence that prenatal programming may have a lasting influence. However, without a cross-fostering design, the relative contributions remain unresolved. Future studies employing cross-fostering designs or artificial rearing with controlled milk intake will be essential to disentangle the prenatal and postnatal drivers of the observed follicle deficits.

This framework reconciles our current findings with the previously reported observation that body weight gain during the non-growing period can enhance cashmere yield by increasing the population of active SHFs. We propose that such nutritional activation operates within a “hard cap” determined by the absolute SHF number established by approximately 6 months of age. For twin-born goats, an inherently lower total SHF density imposes a permanently lower ceiling for cashmere production, even under optimal later-life nutritional management. The negative genetic correlations between fleece weight and litter size traits reported in sheep [37,38] may therefore partly reflect this early-life programming of follicular architecture, rather than direct gene action alone. These insights carry clear practical implications: breeding and management strategies aimed at improving the lifetime cashmere output of multiple-born goats must prioritize nutritional interventions during late gestation and the first six months of life, as this constitutes the sole developmental window during which the foundational endowment of secondary follicles can be favorably influenced.

Several maternal factors that may influence offspring development were not directly measured in this study, including individual milk production, maternal body condition during gestation and lactation, and gestational nutritional intake. These factors may contribute to the observed differences between single- and twin-born kids and represent potential confounding effects that cannot be fully excluded. It should be noted, however, that these maternal differences are themselves integral components of the birth-type contrast under standard production conditions. Twin-bearing ewes inherently face greater metabolic demands, and their reduced capacity to meet these demands is part of the biological pathway through which prolificacy affects offspring development. From an applied perspective, the observed differences reflect the integrated effect of birth type as it operates in commercial practice. Although we have investigated the effects of birth type, we cannot completely rule out the subtle influences of maternal nutrition and intrauterine environmental differences. Future studies should incorporate individual-level measurements of maternal milk yield (e.g., via the weigh–suckle–weigh method), body condition scoring at key time points, and controlled nutritional regimens to partition the effects of birth type per se from those mediated by differential maternal investment.

## 5. Conclusions

The present study reveals a striking tissue-specific developmental dichotomy in cashmere goats: twin-born kids, despite experiencing intrauterine growth restriction and depressed pre-weaning growth, achieved full compensatory somatic growth and reached yearling body weights indistinguishable from those of singletons, yet this plasticity did not extend to the cashmere-producing apparatus. Single-born goats maintained a lasting superiority in greasy cashmere yield and fiber fineness, which was mechanistically underpinned by a selective and irreversible advantage in all secondary hair follicle traits—including higher density, number, and secondary-to-primary follicle ratio—while primary follicle parameters remained entirely unaffected. The strong negative correlation between secondary follicle density and fiber diameter provides a direct histological basis for the observed fiber quality differences. In conclusion, birth type shows a lasting association with secondary hair follicle endowment and cashmere production in yearling goats, with twin-born kids exhibiting lower SFD, SFN, S/P ratio, and cashmere yield despite achieving equivalent body weight. These effects persist at least into early adulthood. Combined with the known developmental timeline of SHFs, our findings suggest that the period from late gestation through the first three months of life may represent a critical window during which follicle endowment is influenced in a manner not fully recoverable through post-weaning nutrition, and that targeted nutritional strategies during this window may offer an opportunity to mitigate the trade-off between prolificacy and fiber production. However, as our study design does not directly establish causality, and the duration of follow-up does not extend beyond yearling age, longer-term studies with controlled nutritional interventions are required to confirm whether these differences represent irreversible developmental programming and whether such strategies are effective.

## Figures and Tables

**Table 1:** Growth performance of single- and twin-born kids during the pre-weaning period.

Item	Single-Born Kid	Twin-Born Kid	*p* Value
Birth weight, kg	2.85 ± 0.06	2.41 ± 0.06	<0.001
Weaning weight, kg	20.17 ± 0.32	18.28 ± 0.17	<0.001
Yearling weight, kg	32.92 ± 0.52	32.88 ± 0.50	0.963
Weight gain during lactation, kg	17.31 ± 0.31	15.85 ± 0.20	<0.001
Weight gain during rearing period, kg	12.76 ± 0.48	14.60 ± 0.48	0.009
ADG during lactation, g/d	146.5 ± 3.4	127.9 ± 1.6	<0.001
ADG during rearing period, g/d	51.8 ± 1.9	60.5 ± 1.9	0.001

Note: Values are least squares means ± standard error of the mean (LSM ± SEM) from a linear mixed model. The model included birth type (single vs. twin) and parity as fixed effects, with dam ID fitted as a random effect to account for the clustering of twin kids born to the same dam. *p* values were obtained from the pairwise comparison of least squares means.

## Data Availability

The raw data supporting the conclusions of this article will be made available by the authors on request.

## Abbreviations

The following abbreviations are used in this manuscript:

ADG	Average daily gain
IUGR	Intrauterine growth restriction
PF	Primary follicle
PFD	Primary follicle density
PFDI	Primary follicle density index
PFN	Primary follicle number
SF	Secondary follicle
SFD	Secondary follicle density
SFDI	Secondary follicle density index
SFN	Secondary follicle number
SHF	Secondary hair follicle
S/P	Ratio of Secondary follicle to primary follicle
